# Physiological and molecular mechanisms of tolerance to hypoxia and oxygen deficiency resistance markers

**DOI:** 10.3389/fmolb.2025.1674608

**Published:** 2025-11-26

**Authors:** Maria Silina, Dzhuliia Dzhalilova, Nikolai Fokichev, Olga Makarova

**Affiliations:** 1 Laboratory of Inflammation Immunomorphology, Avtsyn Research Institute of Human Morphology of Federal State Budgetary Scientific Institution «Petrovsky National Research Centre of Surgery», Moscow, Russia; 2 Department of Hystology, Petrovsky Medical University, Moscow, Russia; 3 Faculty of Biology and Biotechnology, HSE University, Moscow, Russia

**Keywords:** hypoxia, biomarkers, hypoxia tolerance, acute mountain sickness, personalized medicine

## Abstract

Humans and laboratory animals differ in their resistance to hypoxia, which affects the severity of inflammatory diseases and the rate of tumor progression. Therefore, it is necessary to search for methods for assessing the initial tolerance to oxygen deficiency and the risks of developing acute mountain sickness without direct hypoxia exposure on the organism. The literature describes assessments of oxygen deficiency tolerance assessing based on physiological and molecular parameters. The limitations of such methods include the need of expensive equipment use and highly qualified specialists involvement. In addition, in the studies presented in this review, the altitude, time and methods for achieving it, as well as the frequency of assessing the severity of acute mountain sickness varied significantly. It is necessary to continue research aimed at investigating biomarkers of tolerance to oxygen deficiency without direct hypoxia exposure using instrumental and laboratory diagnostics. This will allow developing new approaches for personalized prevention, diagnosis and treatment of inflammatory diseases and malignancies and, accordingly, improve the quality of life.

## 1 Introduction

Hypoxia, or decreased oxygen, is one of the main factors regulating the functional state of the organism both under physiological conditions and in pathology. Depending on the cause, hypoxia is classified as exogenous and endogenous. Exogenous hypoxia develops under the influence of various environmental factors (Shaw et al., 2021). For example, normobaric hypoxia occurs during the prolonged stay in poorly ventilated rooms. In its turn, hypobaric hypoxia is a consequence of the decrease in barometric pressure and, consequently, lower atmospheric pO_2_, which can lead to acute mountain sickness (AMS). Endogenous hypoxia is associated with a particular organ system and disruption of metabolic function. For instance, respiratory hypoxia is associated with pathology of the respiratory organs and ventilation disorders, circulatory hypoxia is associated with the cardiovascular system, hemic (anemic) hypoxia is associated with a decrease in the oxygen capacity of the blood, and tissue (histotoxic) hypoxia is associated with mitochondrial dysfunction. Depending on the duration and type of exposure, acute, chronic, intermittent and interval hypoxia are distinguished (Prabhakar and Semenza, 2012). According to literature, acute hypoxia develops within a few minutes and lasts 1–2 h, after which it transitions into subacute and, ultimately, chronic forms, in which the organism can remain for several days, months or years. Periodic hypoxia occurs due to repeated episodes of respiratory arrest and is most often observed in patients with obstructive sleep apnea syndrome (Santocildes et al., 2021). Interval hypoxia could be used for athletic training and preparing people for high altitude ascents training, which includes the cyclic alternation of hypoxia and reoxygenation (Burtscher et al., 2010).

Systemic hypoxia is observed in severe acute respiratory inflammatory infections and anemic conditions followed by impaired oxygen absorption or transport (Lee H. J. et al., 2019). When the body adapts to oxygen deficiency conditions, blood volume is centralized, leading to a decrease in blood supply in tissues and organs that are more resistant to hypoxia and an increased blood supply to vital ones (Mirna et al., 2020). Such microcirculatory changes cause damage and death of cells and tissues. Moreover, hypoxia-related disorders increase the likelihood of complications and death in severe infectious and inflammatory diseases such as sepsis and acute respiratory distress syndrome, which develops, for example, with COVID-19 infection (Urakov et al., 2022). The severity of inflammatory diseases is influenced by many factors, including individual hypoxia tolerance, which varies appreciably in the human population (Esper et al., 2006; Mayr et al., 2014; Kosyreva et al., 2019; Dzhalilova et al., 2019).

Humans and laboratory animals differ according to their tolerance to hypoxia, which affects the course of inflammatory processes, as well as rates of initiation and progression of malignant tumors (Mayr et al., 2014; Dzhalilova et al., 2019; Dzhalilova et al., 2023; Dzhalilova et al., 2024a; 2024c; 2025; Kirova et al., 2013; Jain et al., 2013; Jain et al., 2014). To determine the oxygen deficiency tolerance in humans, the hypoxia exposure correspondence to several thousand meters altitude (up to 6500 m) is used (Bustamante-Sánchez et al., 2019; Tu et al., 2020; Lu et al., 2018; Kammerer et al., 2018). Since laboratory animals are generally more tolerant to hypoxia in comparison to humans, extremely high altitudes are used to determine their resistance to oxygen deficiency than altitudes used for humans. For C57Bl/6 mice, such a critical “altitude” is 10,000 m (Vedunova et al., 2014), and for Wistar rats – 11,500 m (Kirova et al., 2013). In this case, animals are identified as hypoxia-tolerant or -susceptible by determining the “gaping time”, i.e., the time spent at the simulated altitude before assuming lateral position and displaying signs of asphyxia including gasping and lateral recumbency. The literature also describes markers of AMS found in high-altitude conditions. Such exposure to hypoxia *in vivo* leads to damaging and inflammatory changes in internal organs, which complicates this approach to determining hypoxia resistance in healthy individuals and patients (Makarova et al., 1992). Therefore, to assess the risks of developing pathological conditions during hypoxia, it is necessary to search for biomarkers without the direct exposure to oxygen deficiency.

## 2 Physiological mechanisms of response to hypoxia of organs and tissues and related hypoxia resistance biomarkers

In response to acute hypoxia, various types of compensations develop (Berger and Grocott, 2017). First of all, hypoxia activation of peripheral and central chemoreceptors mobilizes mechanisms that enhance blood oxygen transport. Central chemoreceptors in the medulla oblongata respond to changes in the pCO_2_ and pH intracerebral interstitial fluid and, thus, regulate the depth of inhalation. Peripheral chemoreceptors include carotid and aortic bodies, which respond to the decreased arterial pO_2_ by increasing respiratory frequency (Caballero-Eraso et al., 2023). Hemoglobin’s affinity for oxygen is lower in the periphery (in tissues) and higher in the pulmonary blood vessels. This occurs because the partial pressure of oxygen in tissues decreases, CO_2_ increases, and pH decreases (the Bohr effect (Malte and Lykkeboe, 2018)), which forces hemoglobin to release oxygen. In the lungs, on the contrary, high partial oxygen pressure and low partial CO_2_ pressure promote strong hemoglobin to oxygen binding. During hypoxia, the tone of the vagus nerve decreases and the vasomotor center in the medulla oblongata is excited. This results in an increase in stroke volume and heart rate, as well as a decrease in peripheral vascular resistance (Richalet et al., 2024). The phenomenon of hypoxic pulmonary vasoconstriction is distinguished as one of the mechanisms for increasing oxygen supply to organs. It is based on the Euler-Liljestrand reflex - when the concentration of O_2_ in the alveoli decreases, the blood supply to those alveoli decreases by narrowing the vessels of the pulmonary circulation (Euler and Liljestrand, 1946). At the same time, the vessels of the systemic circulation, on the contrary, expand. As a result, blood flow is centralized, leading to a decrease in blood supply in tissues and organs that are more resistant to hypoxia and an increase in vital ones, which include the brain, heart, liver and kidneys (Mirna et al., 2020). In case of severe hypoxia, due to vasoconstriction of small veins and arteries, the blood reservoirs also are mobilized which increases hematocrit and the numbers of circulating normoblasts and reticulocytes. In a murine model of acute hypoxia (4,500 m, 16 h), it was demonstrated that in the foci of normal and extramedullary stress erythropoiesis, the content of erythroid nucleated cells decreases, especially orthochromatophilic cells ready for enucleation, and the number of lymphoid cells increases (Nazarov et al., 2023).

### 2.1 Renal hemodynamics and integrity of the glomerular filtration membrane

While the hypoxic response of the respiratory and cardiovascular systems are well described in detail in the literature, hypoxia-induced changes in renal function are much less studied. Under normoxic conditions, the main kidneys functions are the regulation of hemodynamics and maintenance of electrolyte balance. Under hypoxia, in response to centralization and increased blood supply, energy expenditure and the kidneys’ need for oxygen increase, which determines the high sensitivity of this organ to oxygen deficiency (Evans et al., 2020). Unlike other organs, kidney adaptation to hypoxia is realized by two mechanisms: synthesis of erythropoietin and control of electrolyte and water excretion. Erythropoietin is secreted mainly by the kidney’s interstitial cells and, to a lesser extent, by the liver’s perisinusoidal cells, while the induction of its expression depends on the HIF-2α (Hypoxia-Inducible Factor 2 alpha) protein, since the *EPO* gene promoter contains the corresponding binding sites for HIF-2α and its coactivators (Keith et al., 2011). Erythropoietin increases the mitotic activity of erythrocyte precursor cells and promotes erythrocyte maturation, thus increasing the blood oxygen-supplying capacity under stress conditions (Lee C.-J. et al., 2019; Montero and Lundby, 2019). Diuresis and natriuresis increase under hypoxic conditions, which reduces the volumes of extracellular fluid and plasma, as well as to an increase in renal blood supply and tubular function (Evans and Bie, 2016). At the same time, *in vitro* and *in vivo* studies demonstrated that HIF-1α protein activation in podocytes indirectly leads to damage to these cells and the development of renal proteinuria (Nakuluri et al., 2019a; 2019b). HIF-1α induces gene expression and synthesis of ZEB2 (Zinc finger E-box-Binding homeobox 2) and TRPC6 (Transient Receptor Potential Canonical 6) proteins, which leads to the decrease in synthesis of the adherens junction protein E-cadherin and nephrin, podocin and other components of podocyte slit diaphragms. As a result, the shape of the podocytes changes, their pedicels become deformed and, as consequence, the filtration function of the kidneys is disrupted (Singh et al., 2021). Thus, under conditions of oxygen deficiency, the kidneys' response is not aimed at protecting the organ from possible damage, but at adapting the organism in general.

### 2.2 Blood brain barrier integrity

Another organ sensitive to hypoxia is the brain. Due to the blood flow centralization, its oxygen supply remains almost unchanged (extreme (voluntary) asphyxia), but hypoxic exposure lasting more than a few minutes leads to a decrease in cerebral blood flow and an increase in the permeability of the blood-brain barrier (BBB) (Bailey et al., 2022). Disruption of the BBB promotes the adhesion and migration of circulating leukocytes into brain tissue, where the invasive leukocytes secrete proinflammatory cytokines which promote neuronal damage and death, and cerebral edema (Jin et al., 2013).

### 2.3 Bone structure and remodeling

In addition to functional disorders in organs sensitive to oxygen deficiency, exposure to hypoxia also results in bone loss, but usually does not cause any obvious symptoms. After a 6-month stay at 2,500 m altitude, a decrease in bone mineral density in the spine is observed (5 male subjects (aged 28–54 years, BMI preexpedition 26.36 ± 3.87 kg/m^2^) were assessed) (O’Brien et al., 2018). After 4 months spent at an altitude of 3,450 m, the activity of alkaline phosphatase, including its bone-specific isoform, and the C-terminal telopeptide of collagen type I decreases, and at 5,400–6,700 m altitude, morphological and structural changes in bone tissue occur (Basu et al., 2013; 2014). Under the oxygen deficiency conditions, HIF-1α binds to HREs (Hypoxia-Response Elements) in the promoter of the *Sost* gene, which encodes sclerostin (Chen et al., 2013). This protein is constitutively synthesized in osteocytes and regulates osteogenesis and osteolysis. By binding the low-density lipoprotein receptors LRP5/6 (Lipoprotein Receptor-related Proteins), sclerostin inhibits the Wnt/β-catenin signaling cascade and the subsequent induction of Runx2 (Runt-related transcription factor 2) and osteoprotegerin synthesis, which promotes the differentiation of progenitor osteogenic cells (Tu et al., 2015; Cai et al., 2016). Experiments on laboratory animals demonstrated that 18 h exposures to 7,500 m simulated altitude in a decompression chamber produced changes in bone tissue structure characterized by decreased strength of the femoral diaphysis, disruption of the trabecular microstructure, and increased tissue contents of sclerostin, the osteoclast differentiation and activation factor RANKL (Receptor Activator of Nuclear Factor Kappa-B Ligand), and proinflammatory cytokines IL-1β and TNF-α in bone tissue (Wang et al., 2020). Exposure on the UMR106.01 rat osteogenic cell line to 96 h of normobaric hypoxia (1% O_2_) vs. normoxia (21% O_2_) decreased *Sost* gene expression and sclerostin content, while activating the Wnt/β-catenin cascade (Genetos et al., 2010). These contradictory results may be due to the different conditions of the experiments. Standard conditions of normoxia *in vitro* are considered to be 21% O_2_, while *in vivo* partial pressure of oxygen varies among the organs and tissues. The effects of different oxygen concentrations on cells *in vitro*, which might not correspond to normoxic and hypoxic conditions *in vivo*, may disturb signaling pathways and, consequently, alter the cellular phenotype (Prabhakar and Semenza, 2012), which causes the contradictory results.

### 2.4 Gut microbiome and intestinal epithelial barrier function

The intestinal microbiome plays an important role in the human body under hypoxic conditions (Kumar et al., 2020). Colonic clusters of *Firmicutes*, *Bacteroidetes*, and *Clostridial* bacteria convert dietary fiber and resistant starch into short-chain fatty acids (SCFA). Bacteria activate PDK (Phosphoinositide-Dependent Kinase) in enterocytes, which promotes oxidation of SCFA and the formation of acetyl-CoA and its inclusion in the tricarboxylic acid cycle (Parada Venegas et al., 2019). However, increased SCFA oxidation increases O_2_ consumption which leads to physiological hypoxia and HIF proteins stabilization (Kelly et al., 2015). It was demonstrated that HIF-1α stabilization induces gene expression and synthesis of the membrane protein of tight junctions proteins claudin-1 (CLDN1), Junctional Adhesion Molecule-A (JAM-A) and occludin, which support the structure and functions of tight junctions between enterocytes (Van Itallie and Anderson, 2014; Muenchau et al., 2019). The mucous layer secreted by intestinal goblet cells forms a barrier that limits direct contact of pathogens with the intestinal epithelium (Johansson et al., 2008). Its main components are mucin proteins, among which MUC2 predominates (Holmén Larsson et al., 2013). Activation of HIF-1α leads to increased content of MUC2, MUC3 and Intestinal Trefoil Factor (ITF) in mucus, enhancing the barrier function of the mucous layer (Louis et al., 2006; Dilly et al., 2016; Glover et al., 2016).

### 2.5 Physiological markers of hypoxia resistance

Currently, hypoxia tolerance of healthy humans is determined by exposure to hypoxia corresponding to an altitude of several thousand meters (Bustamante-Sánchez et al., 2019; Tu et al., 2020; Lu et al., 2018; Kammerer et al., 2018). However, such an analysis was only performed on astronauts and supersonic pilots, i.e., on a limited number of highly fit people, and sensitivity to oxygen deficiency was assessed using the LLS (Lake Louise Scale), as in the diagnosis of AMS. This scale is based on a self-assessment of headache, gastrointestinal symptoms, fatigue or weakness, dizziness, and insomnia. People with a total LLS score of ≥3 with severe headache after ascent to a high altitude environment are considered hypoxia-susceptible to the develop AMS, while people with a total LLS score of ≤2 or without headache after exposure to hypobaric hypoxia, are defined as hypoxia-tolerant (Kammerer et al., 2018; Lu et al., 2018; Roach et al., 2018). The LLS index is considered to be a rather subjective method for assessing the AMS severity prognosis (Roach et al., 2018). Over the last 5 years, several instrumental approaches for determining in advance the risks of AMS development before ascent to altitude were described in the literature (Supplementary Table S1).

Hypoxia physiological and pathological effects studies are conducted in hypobaric conditions either at simulating high altitude, or in normobaric conditions at reduced atmospheric O_2_ level, which are easier to modulate in an experiment. However, the oxygen partial pressure under such conditions might not be the only altered variable. Thus, in human subjects hypobaric hypoxia exposure lowered minute and alveolar ventilation, worsened AMS symptoms based on the LLS, provoked postural instability, lowered NO levels in the expired air and systemic circulation, and increased oxidative stress vs. normoxic exposures (Coppel et al., 2015). Thereafter, to assess the risks of developing AMS, it is necessary to use hypobaric hypoxia, with high altitude conditions replicates better than normobaric hypoxia. Adaptation to hypoxia depends not only on the mode and duration of the hypoxic exposure, but also on the initial organism tolerance to oxygen deficiency. Therefore, identifying the biomarkers of hypoxia tolerance is a focus of current high altitude research.

The study (Ke et al., 2021) included 106 men aged 20.0 ± 3.0 years who performed step-by-step ascent by bus for 7 days (about 40 h of ascent and about 40 h of rest and sleep without altitude change) from 400 m to 4,100 m. These men were diagnosed with AMS according to the LLS scale once upon reaching altitude, after which the AMS+ (n = 33) and AMS- (n = 73) groups were identified. Using speckle-tracking echocardiography–natural acoustic echoes analysis set from points with stable visualization during systole and diastole, authors demonstrated that lateral Mitral Valve Tissue Motion Annular Displacement (MV TMADlateral) reflects longitudinal systolic function and with relative weakness can be the AMS predictor. However, this method, according to the authors, is characterized by the low sensitivity and specificity - 72.7% and 60.3%, respectively.

In a number of studies, maximum oxygen consumption (VO_2max_) and blood oxygen saturation (SpO_2_) were characterized as markers of tolerance to AMS (Seiler et al., 2023; Ye et al., 2023; Zeng et al., 2024; Joyce et al., 2024). These studies included healthy male and female volunteers aged 20 to 60, and measurements were taken using both classical methods (cardiopulmonary exercise test and pulse oximetry) and smartwatches. It was demonstrated that VO_2max_ and SpO_2_ were statistically significantly lower in people categorized as AMS+ (Ye et al., 2023; Zeng et al., 2024), with VO_2max_ below 49.0 mL/min/kg before ascent correlating with the AMS development during the ascent without the use of additional oxygen (Seiler et al., 2023), and the increase in SpO_2_ by one unit reduces the risk of AMS by 9% (Zeng et al., 2024). In addition, overnight SpO_2_ values at 3,850 m correlate with AMS at 4,800 m (Joyce et al., 2024). These markers also demonstrated the lack of sensitivity and specificity. Furthermore, the studies did not take into account sex differences, which were previously demonstrated to influence tolerance to low oxygen levels as well (Dzhalilova et al., 2020).

The studies by Zhang H. et al. and Zhang W. et al. also included healthy male and female volunteers (in a ratio of approximately 1:1), and the identified markers of resistance to AMS were associated with cerebral blood flow velocity and had good diagnostic value (Zhang H. et al., 2024; Zhang W. et al., 2024). Using brain MRI, the fractional amplitude of low-frequency fluctuations (fALFF) and the degree centrality from resting-state functional MRI, mainly distributed in the somatomotor network, were identified as predictors of AMS. Before the ascent, these were statistically significantly lower in the AMS- group compared to the AMS+ group (Zhang W. et al., 2024). In a study using three-dimensional pseudo-continuous arterial spin labeling (3D-pCASL), the authors demonstrated that higher cortical cerebral blood flow in the right posterior cerebral artery before the ascent correlated with AMS+ in men, while laterality index of cerebral blood flow in the anterior cerebral artery correlated with AMS+ in women (Zhang H. et al., 2024).

Despite the differences between the groups of people with and without AMS identified before the ascent, most of the presented instrumental studies require expensive equipment and highly qualified specialists. A significant limitation of these tests is their low diagnostic sensitivity and specificity. In studies using smart watches, the measured parameters are not validated or standardized, which reduces their value of determining AMS risk. In addition, the time, ascent height and methods for achieving it, as well as the frequency of AMS diagnostics, differ amongst published reports, which undoubtedly affected the results of the study. Despite the similar age of studied populations, the lack of differentiated assessment of the men and women in most published reports may have limited study reliability, since experiments on laboratory animals demonstrated that females were more resistant to hypoxia than males (Dzhalilova et al., 2020).

## 3 Molecular mechanisms of response to hypoxia and markers of tolerance to oxygen deficiency

**FIGURE 1:**
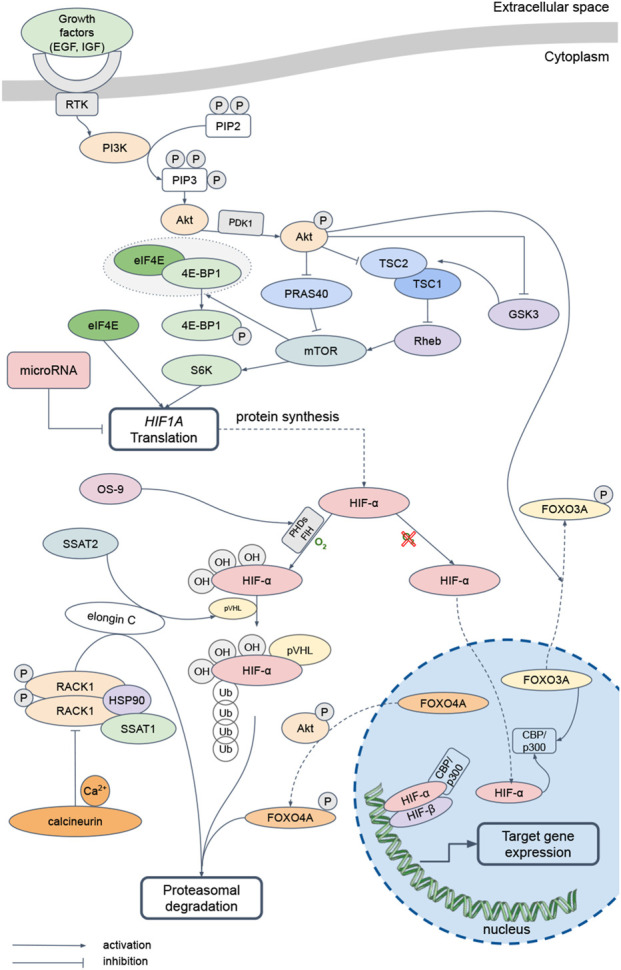
The main pathways regulating HIF-α-subunits translation and activity. EGF – Epidermal Growth Factor, IGF – Insulin-like Growth Factor, RTK – Receptor Tyrosine Kinases, PI3K – Phosphatidylinositol-3-Kinase и Protein kinase B alpha, PIP2 – Phosphatidylinositol 4,5-bisphosphate, PIP3 – Phosphatidylinositol (3,4,5)-trisphosphate, PDK1 – Phosphoinositide-Dependent Kinase, TSC1 – Tuberous Sclerosis Complex 1, TSC2 – Tuberous Sclerosis Complex 2, GSK3 – Glycogen Synthase Kinase-3, Rheb – Ras homolog enriched in brain, PRAS40 – Proline-Rich Akt substrate 40 kDa, mTOR – mammalian Target of Rapamycin, eIF4E – Eukaryotic Initiation Factor-4E, 4E-BP1 – Eukaryotic Initiation Factor-4E Binding Protein 1, S6K – p70 ribosomal protein S6 Kinase, HIF – Hypoxia-Inducible Factor, PHDs – Prolyl-Hydroxylases, FIH – Factor-Inhibiting HIF-1α, OS9 – Osteosarcoma amplified 9, SSAT1 – Spermidine/Spermine-N1-Acetyltransferase 1, SSAT2 – Spermidine/Spermine-N1-Acetyltransferase 2, pVHL – protein von Hippel-Lindau, RACK1 – Receptor of Activated Protein Kinase C, HSP90 – Heat Shock Protein 90, FOXO3A – Forkhead box O protein 3 А, FOXO4A – Forkhead box O protein 4 A, CBP/p300 – CREB (cAMP-Response Element-Binding protein)-Binding Protein/E1A Binding Protein p300

The Figure 1 diagrams the signaling pathways that regulate translation and activity of HIF-α subunits. The HIF family of transcription factors are the main regulators of the cellular response to hypoxia. The synthesis of these proteins depends on mTOR, the activation of which is associated with the PI3K-Akt signaling cascade (Badoiu et al., 2023). When various growth factors, including EGF and IGF, interact with the corresponding receptors, PI3K phosphorylates of phosphatidylinositol and phosphoinositides, which then bind Akt to facilitate its phosphorylation by PDK1. Further signal transmission from activated Akt to mTOR occurs in several ways. One of them is Akt-dependent phosphorylation and inhibition of tuberin, or TSC2 (Dibble and Cantley, 2015). It forms a complex with hamartin, or TSC1, and the inactive GTPase Rheb. During Akt-dependent phosphorylation of tuberin, Rheb is not inhibited and activates mTOR. Akt can also phosphorylate PRAS40, permitting increased mTOR activity (Laplante and Sabatini, 2012). Then mTOR phosphorylates 4E-BP1, permitting ribosomal complex eIF4E (Yang M. et al., 2022). mTOR also phosphorylates S6K, promoting translation of mRNAs containing the 5′-TOP region in their 5′-untranslated region. Thus, translation of *HIF1A* mRNA is regulated by a PI3K-Akt-mTOR-dependent pathway.

The three mammalian isoforms, HIF-1α, HIF-2α, HIF-3α, combine with the constitutively expressed HIF-1β subunit (Semenza, 2001) forming the transcriptionally active HIF heterodimers. The subunits are degraded under normoxic conditions, but stabilized by hypoxia in proportion to its severity (Monaci et al., 2024). In the presence of oxygen, PHDs hydroxylate proline residues at positions 564, 530, and 490 in HIF-1α, HIF-2α, and HIF-3α, respectively (Haase, 2017). The hydroxylated subunits then undergo ubiquitination in the presence of the pVHL (Jaakkola et al., 2001). Also, FIH hydroxylates arginine residues at positions 803 and 851 in HIF-1α and HIF-2α, which disrupts the binding of these proteins to the coactivator CBP/p300 and leads to a decrease in the HIF transcriptional activity. Hypoxic conditions limit hydroxylation of the HIF subunit, which translocates into the nucleus, where it forms a complex with HIF-1β and CBP/p300 and activates the transcription of dependent genes (Burtscher et al., 2021). In addition, short-term acute exposure to oxygen deficiency (up to 60 min, 1%–3% O_2_), increases activity of the PI3K/Akt pathway, which leads to inhibition of GSK3, which in this modification does not regulate TSC1 and TSC2 and, thus, activates translation (Yee Koh et al., 2008).

HIF subunits also are regulated by an oxygen-independent pathway. Thus, the OS-9, by binding to PHD2 or PHD3, promotes the HIF α-subunit hydroxylation (Baek et al., 2005). Then, SSAT2 forms a complex with pVHL and elongin C, which stabilizes the proteasome to degrade HIF-α regardless of the oxygen concentration (Baek et al., 2007). Furthermore, elongin C can bind to the homodimer of phosphorylated protein RACK1, which, in complex with heat shock protein HSP90 and SSAT1, promotes HIF α-subunit ubiquitination and proteasomal degradation independently of oxygen and pVHL (Liu et al., 2007). Calcium and calcineurin inhibit the RACK1 homodimer formation and, thus, participate in the HIF α-subunit degradation regulation (Lee H. J. et al., 2019). PI3K-Akt signaling pathway affects HIF α-subunit translation and stabilization. Akt phosphorylates transcription factors FOXO3A and FOXO4A, which promotes their translocation from the nucleus to the cytoplasm. Then, phosphorylated FOXO4A promotes ubiquitination and proteasomal degradation of HIF-α in a pVHL-independent pathway (Wang et al., 2021). At the same time, dephosphorylated FOXO3A binds the coactivator CBP/p300, thereby blocking the formation of HIF heterodimers and the induction of target gene expression (Emerling et al., 2008). GSK3 also negatively regulates HIF-α by phosphorylating its oxygen-dependent degradation and N-terminal transactivation domains, thereby promoting HIF ubiquitination and degradation (Kietzmann et al., 2016).

Activation of the HIF α-subunit isoforms also depends on the hypoxic exposure duration. In various human cell lines HIF-1α regulates the response to acute oxygen deficiency (cell incubation for less than 2 h at an O_2_ concentration of 0.5%), while HIF-2α regulates the response to long-term oxygen deficiency (cell incubation for more than 14 h at an O_2_ concentration of 0.5%), with HIF-3α and some microRNAs responsible for isoform switching (Moszyńska et al., 2022). MicroRNAs are non-coding RNA molecules consisting of 18–25 nucleotides that regulates gene expression by preventing translation or destroying mRNA. They are simultaneously capable of regulating a large number of target mRNAs and thus influencing the activation of several signaling pathways that control protein synthesis. To date, more than 100 microRNAs involved in the regulation of the HIF-mediated response to hypoxia have characterized. Among them are miR-210, activated by HIF-1α and affecting the proteins involved in metabolic adaptation to hypoxia synthesis (Fasanaro et al., 2008), miR-107, regulating HIF-1β (Yamakuchi et al., 2010), miR-145, inhibiting HIF-2α synthesis (Zhang et al., 2014), miR-429, implementing the HIF-1α and HIF-3α isoforms switching (Janaszak-Jasiecka et al., 2016), and miR-155, the most active regulatory microRNA in the liver (Yang et al., 2016). More details about HIF-α isoforms and the microRNA role in the regulation of the cellular response to hypoxia were previously described in our review (Silina et al., 2023).

### 3.1 HIF-dependent mechanisms of cellular response to hypoxia

More than 1,000 molecules implementing the cellular response to oxygen deficiency are known targets for HIF transcription factors. Although the genes regulated by HIF-1α and HIF-2α may overlap, numerous studies demonstrated that these isoforms have different target genes. Among the common HIF-1α and HIF-2α targets are the vascular endothelial growth factor *VEGFA* and the glucose transporter 1 *GLUT1* (Loboda et al., 2010).

Among the HIF-α isoforms, only HIF-1α activates transcription of genes encoding glycolytic enzymes. Under hypoxic conditions, this isoform, by binding to HREs, induces the gene encoding the PDK1 enzyme expression, which inhibits mitochondrial pyruvate dehydrogenase and, thus, prevents the pyruvate to acetyl-CoA conversion and the entry of the latter into the tricarboxylic acid cycle (Papandreou et al., 2006). As a result, there is a transition from oxidative to glycolytic metabolism, a decrease in ATP synthesis and the reactive oxygen species (ROS) production. The cellular energy deficit is compensated by inducing the expression and synthesis of glucose transporters GLUT1 and GLUT3, which are also HIF-1α targets (Kalinowski et al., 2016). In addition, HIF-1 activates the production of glycolytic enzymes as hexokinase, phosphofructokinase, aldolase, glyceraldehyde-3-phosphate dehydrogenase, phosphoglycerate kinase, enolase and pyruvate kinase (Taylor and Scholz, 2022). HIF-1α also enhances expression and synthesis of lactate dehydrogenase, which converts pyruvate to lactate with conversion of NADH to NAD+, which is necessary for glycolysis, and monocarboxylate transporter 4, which exports the lactate from the cell (Ullah et al., 2006).

At the same time, erythropoiesis and iron metabolism largely depend on HIF-2α. The *EPO* gene promoter contains binding sites for this isoform which, under hypoxic conditions, activates erythropoietin synthesis in the kidneys and liver (Rankin et al., 2007). Moreover, one of the HIF-2α targets is the *DMT1* gene, encoding the iron import protein on the apical membrane of enterocytes (Shah et al., 2009). Depending on the organism’s needs, iron can either be deposited in a complex with ferritin or exported through the enterocyte’s basal membrane into the plasma using ferroportin. Thus, HIF-2α contributes to the cellular response to oxygen deficiency formation, regulating erythropoiesis and iron metabolism (Keith et al., 2011; Mastrogiannaki et al., 2009).

### 3.2 HIF-independent cellular response to hypoxia mechanisms

HIF-independent mechanisms are also involved in the cellular response to oxygen deficiency, which include post-translational modifications, spatial reorganization and the allosteric regulation of glycolytic enzymes activity and epigenetic reprogramming (Lee et al., 2021).

Fructose-2,6-bisphosphate acts as allosteric regulator of glycolysis and gluconeogenesis (Ros and Schulze, 2013). When it binds to the corresponding site on the phosphofructokinase-1 molecule, the affinity of the enzyme for fructose-6-phosphate increases and the affinity for ATP and citrate, which are the reaction inhibitors, decreases. Thus, fructose-2,6-bisphosphate stimulates glycolysis in the liver, inhibits fructose-1,6-bisphosphatase and slows down gluconeogenesis. This allows cells to optimize metabolism and adapt to hypoxic conditions.

Glycolytic enzymes are organized into multi-enzyme granules termed G-bodies. A study on *Saccharomyces cerevisiae* and the human hepatocarcinoma cell line HepG2 demonstrated that the formation of such G-bodies can increase the rate of glycolysis, thereby regulating cellular metabolism and adaptation to oxygen deficiency (Jin et al., 2017). Several mechanisms explaining the effect of G-bodies on the increase in glycolytic flux are presented in the literature. These include decreased substrate inhibition by phase separation, substrate channeling by intermediate metabolites concentration, and glycolytic enzymes enhanced translation by concentrating glycolytic enzymes with their cognate mRNAs (Lee et al., 2021). These results suggest that G-body formation is a highly conserved adaptive response that enhances glycolytic processes during hypoxia.

Epigenetic reprogramming is a heritable change in gene expression that is not associated with the DNA sequence alteration and is dependent on environmental exposures (Peixoto et al., 2020). The best-currently studied epigenetic mechanism is methylation of cytosine bases in DNA, which is catalyzed by DNMT1, DNMT3a, and DNMT3b (DNA-Methyltransferase) and, in most cases, silences genes. Among histone modifications, the most common are acetylation of lysine residues at positions 9 and 14 and trimethylation of the same amino acid at positions 27 and 36 of histone H3 (H3K9ac, H3K14ac, H3K27me3, and H3K36me3, respectively). Acetylation and methylation neutralizes lysine’s cationic charge, which disrupts DNA binding by the histone. The resultant detachment of histones opens binding sites for transcription factors. Hypoxic conditions inactivate the lysine methylases and, thereby, preserve H3K36me3 and H3K27me3 methylations, allowing for the induction of genes that regulate the cellular response to oxygen deficiency, including *HIF1A* (Chakraborty et al., 2019; Batie et al., 2019; Dobrynin et al., 2017).

### 3.3 Molecular markers of hypoxia tolerance

Analysis of biological fluids, especially blood and urine, provides a minimally invasive means of diagnosing a patient’s health status. In recent years, clinical, biochemical, immunological and molecular-genetic tests of blood and its components have became routine procedures. Below are studies focused at finding biomarkers of hypoxia tolerance, based on the cellular response to oxygen deficiency molecular features (Supplementary Table S2).

Hypoxia resistance may be influenced by polymorphisms in genes regulating the cellular response to oxygen deficiency. A study of Zhang et al. involved 604 men aged 18–45 who were ascended by airplane for 2 h from 500 m to 3,700 m (Zhang et al., 2020). After 18–24 h, 320 of them were diagnosed with AMS (AMS + group), while 284 demonstrated no signs of a response to oxygen deficiency. Subsequently, single nucleotide polymorphisms (SNPs) rs675666667 in *EPAS1*, rs3025039 in *VEGFA*, rs7292407 in *PPARA*, and rs2153364 in *EGLN1* were determined in all study participants using MALDI-TOF MS. Each of these SNPs was shown to be associated with the AMS symptoms occurrence of varying severity in different organ systems. SNP rs675666667 (genotype GG) is associated with a higher risk of mild AMS and mild gastrointestinal symptoms, and rs3025039 (genotype CC) is associated with a lower risk of mild AMS and mild headaches. Despite the identified differences, using SNPs to determine resistance to AMS and hypoxia is difficult because SNP marker allele frequencies differ in different populations (Salisbury et al., 2003).

Bioinformatics technologies and machine learning have recently made significant advances, allowing scientists to create various mathematical models for predicting disease progression and early identification of high-risk patients. Yang et al. published one of the first such comprehensive studies (Yang J. et al., 2022). Using proximity extension assay technology (based on the DNA-labeled antibody with a protein in the test liquid interaction, washing and subsequent amplification of DNA by PCR), Multiple Reaction Monitoring technology (based on mass spectrometry with stepwise peptide selection), and machine learning XGBoost, they demonstrated that a combination of phosphoglycerate dehydrogenase, ubiquitin-like modifier activating enzyme 1, ribokinase, guanine nucleotide-binding protein subunit alpha-13, insulin-like growth factor-binding protein 7, ficolin-2, carbonic anhydrase II, and V-set and immunoglobulin domain containing 4 contents in plasma reflects resistance to AMS. Furthermore, plasma phosphoglycerate dehydrogenase content before ascension is 4 times more significant in this AMS prediction model. Guo et al. conducted a study examining adaptation to high-altitude conditions: 40 men aged 21–27 performed ascent by car from 1,400 m to 3,700 m, adaptation for 7 days without changing altitude, and ascent by car for 10 h to 5,000 m (Guo et al., 2023). AMS was diagnosed 36–48 h after reaching 3,700 m and 5,050 m using LLS. Subjects who developed AMS at 3,700 m and 5,000 m were considered the group with severe AMS, and only those at 5,000 m were considered the group with a moderate course. Among the study participants, 8 had severe AMS symptoms, 12 had moderate symptoms, and 20 had no symptoms. Combination of Serum Amyloid P-component (SAP), Alpha-1-Antitrypsin (AAT), Lactotransferrin (LT) content in plasma before ascension was higher in the severe AMS + group in comparison to the AMS- group, combination of SAP and HSP90-α – in the group with moderate course of AMS+ in comparison to AMS-, and combination of SAP and LT – in the group with severe course of AMS+ in comparison to moderate AMS+.

Yang et al. conducted two parallel studies: ascent by car for 2 h from 50 m to 4,300 m and ascent in a decompression chamber for 15 min from 50 m to 4,300 m (Yang et al., 2024). Despite the different conditions, the combination of *HLA-DQB1*, *LOC101927999*, *GAS6*, and *TNNT1* mRNA expression levels in peripheral blood mononuclear cells demonstrated high sensitivity (100%) and specificity (83%) for identifying patients at high risk of developing AMS at high altitude. Thus, the mRNA expression levels of *HLA-DQB1*, *LOC101927999*, and *GAS6* in peripheral blood mononuclear cells before ascension were higher in the AMS- group, and *TNNT1* was higher in the AMS + group.

Li et al. conducted a comprehensive study involving healthy male volunteers using different approaches – systolic blood pressure measurement, forced expiratory volume in one second, peak expiratory flow, forced vital capacity, as well as proteomic and metabolomic analysis (Li et al., 2025). Systolic blood pressure, peak expiratory flow, Acyl-CoA Synthetase long-chain family member 4, Immunoglobulin Kappa Variable 1D-16, Poliovirus Receptor, calcitriol, 2-methyl-1,3-cyclohexadiene, 4-acetamido-2-amino-6-nitrotoluene and multimerin-2 in plasma before ascending were higher in the AMS- group, and prosaposin, 20-hydroxy-prostaglandin E2 and coagulation factor XIII B subunit - in the AMS + group. The combination of these indicators is characterized by high sensitivity and specificity (94% and 91%, respectively), however requires the use of complex and expensive research methods and highly qualified staff.

Molecular markers have high diagnostic sensitivity and specificity. At the same time, sensitivity and specificity of diagnostic tests can be improved by optimizing the measured parameters and applying them to machine learning algorithms. Combined analysis of circulating microRNAs hsa-miR-369-3p, hsa-miR-449b-3p and hsa-miR-136-3p in peripheral blood has proven to be a valid method for assessing AMS risk (Liu et al., 2017). This combination is characterized by a sensitivity of 92.7% and a specificity of 93.5%, which demonstrates high accuracy in predicting the development of AMS during ascent to altitude. Several experimental studies were focused to these microRNAs role in cellular responses to hypoxia and inflammation. Thus, in macrophages obtained from the bone marrow of C57Bl/6 mice, miR-369-3p inhibits the translation of the CCAAT/enhancer binding protein β protein leading to decreased expression and synthesis of its targets - TNF-α and IL-6 (Galleggiante et al., 2019). Later, these researchers demonstrated that an increase in this microRNA expression leads to a decrease in synthesis and activity of iNOS and production of proinflammatory cytokines IL-12, IL-1α, IL-1β and an increase in anti-inflammatory IL-10 and IL-1RA (Scalavino et al., 2020). In addition, on the RAW264.7 cell line, it was revealed that miR-369 inhibits expression of the PSMB9/LMP2 immunoproteasome subunit (Proteasome 20S Subunit Beta 9/Low-Molecular-weight Protein), modulating formation of the complex (Scalavino et al., 2022; 2023). The genes encoding NF-κB inhibitor proteins IKKβ (Inhibitor of Nuclear Factor kappa-B Kinase subunit beta) and A20 3′-untranslated regions were characterized as targets for miR-136. In the spinal cord injury model in Sprague-Dawley rats increased expression of miR-136-5p promotes the formation of IL-1β, IL-6, TNF-α, IFN-α, IKKβ and NF-κB, but suppresses A20 synthesis, leading to pronounced infiltration of inflammatory cells which aggravate spinal cord damage (Deng et al., 2018).

These studies have a number of limitations. As in the studies aimed at developing approaches to determining tolerance to oxygen deficiency based on physiological parameters, the altitude, time and methods of achieving it, as well as the frequency of the AMS severity assessing varied significantly in the methods described above. Moreover, almost all studies were performed with the participation of only men aged 17–35 years, which imposes serious limitations on the use of the identified biomarkers in widespread practice, e.g., in women and the elderly. It is worth noting that the interconnection of some proteins, genes and microRNAs with the cellular response to oxygen deficiency is not well characterized, which complicates their use as predictors, since the mechanism of their regulation by hypoxia remains unclear. Because the frequencies of SNPs differ in different populations (Salisbury et al., 2003), correlating SNPs with environmental conditions impacting the individual, including hypoxia, might not be generalizable to all populations. Consequently, a diagnostically important SNP for one ethnic group may not have prognostic significance for another.

## 4 Perspectives

In the studies described above, the search for hypoxia tolerance markers was conducted before ascent to altitude, but AMS was diagnosed in high altitude conditions. Identification of hypoxia tolerance biomarkers requires objective assessment of the organism’s response. This assessment requires standardization of the hypoxic load - the altitude, duration of stay, and atmospheric oxygen content. Such conditions can be reproduced in experiments on laboratory animals. In addition, when ascending to altitude, the levels of proinflammatory cytokines increase, which identifies them as potential biomarkers of hypoxia resistance.

We conducted studies aimed at finding hypoxia tolerance biomarkers and severe systemic inflammatory response predictors in animals with different tolerances to hypoxia. Against the background of a systemic inflammatory response induced by lipopolysaccharide injection, only hypoxia-tolerant animals demonstrated a decrease in spontaneous and complex mitogen-stimulated (lipopolysaccharide, phytohemagglutinin, and concanavalin A at concentrations of 2, 4, and 4 μg/mL) IL-1β and spontaneous IL-10 production was observed (Kosyreva et al., 2023). The IL-1β/IL-10 ratio during the systemic inflammatory response decreased only in susceptible rats, while no changes in this indicator were observed in tolerant animals. The data indicate the high proinflammatory potential of leukocytes in hypoxia-susceptible rats, which apparently determines a more severe systemic inflammatory response. When analyzing the production of proinflammatory cytokines IL-6 and TNF-α and anti-inflammatory IL-10 by leukocytes under the same incubation conditions in hypoxia-susceptible animals, spontaneous production of all three cytokines was significantly higher, and after stimulation, the IL-1β level more than doubled (Dzhalilova et al., 2024b). Animals with spontaneous production of IL-10 > 50 pg/mL, IL-6>10 pg/mL and TNF-α>10 pg/mL, as well as with an increase in IL-1β production by more than 2-fold upon stimulation were classified as hypoxia-susceptible, while animals with IL-10 < 15 pg/mL, IL-6<9 pg/mL and TNF-α<7 pg/mL and the absence of an increase in IL-1β production upon stimulation, were classified as hypoxia-tolerant. Accordingly, the spontaneous and stimulated cytokine production can serve as non-invasive biomarkers for determining hypoxia tolerance.

In another study, we assessed spontaneous and hypoxia- and mitogen-stimulated IL-1β and IL-10 production by peripheral blood cells of adult male Wistar rats 2 weeks before and 1 month after exposure to sublethal hypoxia in decompression chamber to determine their tolerance to oxygen deficiency (Dzhalilova et al., 2024d). To study spontaneous cytokine production, heparinized blood cells were incubated in a culture medium for 1 and 24 h (37 °C, 21% O_2_ and 5% CO_2_), in a culture medium stimulated by hypoxia for 1 h (37 °C, 1% O_2_ and 5% CO_2_), and in a medium stimulated by a complex mitogen with lipopolysaccharide, phytohemagglutinin and concanavalin A for 24 h (37 °C, 21% O_2_ and 5% CO_2_). The proinflammatory cytokine IL-1β and the anti-inflammatory cytokine IL-10 content in the culture fluid was assessed by ELISA. The response to hypoxic stimulation and exposure to a complex mitogen after sublethal hypoxic stress diverges in hypoxia-tolerant vs. -susceptible animals. Regardless of hypoxia tolerance, under hypoxic exposure and stimulation with a complex mitogen, the production of proinflammatory cytokine IL-1β increases, but in susceptible rats, the production of anti-inflammatory IL-10 decreases, while in tolerant rats it does not change, indicating development of proinflammatory phenotype in hypoxia-susceptible animals after a sublethal hypoxic exposure.

Studies on animals with different tolerance to hypoxia demonstrated that it affects the tumor progression rate and the inflammation severity (Mayr et al., 2014; Dzhalilova et al., 2019; 2023; Dzhalilova et al., 2024a; Dzhalilova et al., 2024c; Dzhalilova et al., 2025; Kirova et al., 2013; Jain et al., 2013; Jain et al., 2014). Therefore, LPS-induced systemic inflammatory response in hypoxia-susceptible animals is characterized by more pronounced both pro- and anti-inflammatory reactions, as well as higher levels of *Hif1a* and *Vegf* mRNA expression in the liver in comparison to hypoxia-tolerant ones (Dzhalilova et al., 2019). Using glioblastoma 101.8 and Lewis lung carcinoma models, it was demonstrated that the systemic inflammation accompanying tumor growth severity is also higher in hypoxia-susceptible rats and mice (Dzhalilova et al., 2023; Dzhalilova et al., 2024c). A study of the initiation and progression of colitis-associated colorectal cancer (CAC) in C57Bl/6 mice with different hypoxia tolerances demonstrated that the incidence of tumor development in the distal colon and their area are higher in hypoxia-susceptible mice in comparison to hypoxia-tolerant ones (Dzhalilova et al., 2024a; Dzhalilova et al., 2025). In addition, *Hif3a*, *Vegf*, *Tnfa*, *Il10*, *Tgfb*, *Cmet*, *Egf*, *Egfr*, *Bax*, *Muc1*, and *Cldn7* higher expression was observed in hypoxia-susceptible animals’ tumors, while only *Egf* was expressed in the peritumoral zone. This indicates the later tumor progression stage in these animals, active migration of immune cells to the tumor growth zone, and activation of a negative feedback mechanism to reduce the high level of proinflammatory molecules (Dzhalilova et al., 2024a). Furthermore, differences in the immune system and inflammatory response were revealed in animals with different hypoxia tolerance in CAC (Dzhalilova et al., 2025). Hypoxia-susceptible mice exhibit more pronounced changes in the subpopulation composition of peripheral blood lymphocytes (increased numbers of cytotoxic T lymphocytes and B lymphocytes), an elevated neutrophil-to-lymphocyte ratio, dilation of the germinal centers of splenic lymphoid follicles, an increase in the absolute and relative numbers of B lymphocytes and NK cells, an increase in the absolute number of cytotoxic T lymphocytes, and a decrease in the relative number of macrophages in the mesenteric lymph nodes. This reflects more pronounced antigen stimulation, as well as immune activation in response to tumor progression. At the same time, hypoxia-tolerant mice exhibit hyperplasia of the thymic cortex, reflecting the chronic colitis active course.

Thus, hypoxia-susceptible organisms are at high risk for developing inflammatory diseases and tumors. It is necessary to search for resistance to oxygen deficiency markers to develop new methods of prevention and personalized therapy for patients. Hypoxia is believed to impair physiological functions and limit performance, but there is evidence that exposure to chronic (hypobaric) hypoxia may be beneficial in some diseases (Nisar et al., 2025). For instance, some studies indicate that residents of high altitudes have a reduced incidence of malignant tumors (Thiersch et al., 2017; Thiersch and Swenson, 2018). Further research is needed to identify specific effects of hypoxia, such as whether cancer risks differ between populations living at high altitude for generations and those who moved to sea level (Calderón-Gerstein and Torres-Samaniego, 2021; Yu et al., 2022).

## 5 Conclusion

Hypoxia tolerance in humans and laboratory animals determines the developing risk and the severity of inflammatory diseases and malignant tumors. Therefore, it is necessary to identify means of assessing the initial tolerance to oxygen deficiency and the risks of developing AMS, without direct exposure to hypoxia. The literature presents approaches to assessing tolerance to oxygen deficiency based on physiological and molecular parameters.

The physiological response to oxygen deficiency is characterized by change in the frequency and depth of breathing, blood flow centralization, increased erythropoiesis, and diuresis. Instrumental-based research methods - ECG, MRI, pulse oximetry, as well as the use of smart watches, identified VO_2max_, SpO_2_, blood flow velocity in the cerebral arteries, as well as the low-frequency fluctuations fractional amplitude and the degree of centrality in functional MRI at rest, mainly distributed in the somatomotor network, as pre-ascent predictors of AMS.

The molecular response to oxygen deficiency is effected by HIF transcription factors and the induction of expression of their target genes. Common HIF-1ɑ and HIF-2ɑ targets include *GLUT1* and *VEGF*. HIF-1ɑ regulates glycolysis, and HIF-2ɑ regulates erythropoiesis and iron metabolism. Using methods such as qRT-PCR, RNA-Seq, MALDI-TOF MS, LC-MS, ELISA and various machine learning models, it was demonstrated that the predictors of AMS before mountain climbing are microRNAs (hsa-miR-134-3p, hsa-miR-15b-5p, hsa-miR-369-3p, hsa-miR-449b-3p and hsa-miR-136-3p), genes (*HLA-DQB1, LOC101927999, GAS6* and *TNNT1*), proteins (PHGDH, UBA1, RBKS, GNA13, IGFBP7, FCN2, CA2, VSIG4, SAP, AAT, LT, HSP90-α, ACSL4, IGKV1D-16, F13B, PSAP, PVR and MMRN2) and metabolites (Uric acid, 2-Methyl-1,3-cyclohexadiene, calcitriol, 4-Acetamido-2-amino-6-nitrotoluene, 20-Hydroxy-PGE2), although their contributions to the cellular response to hypoxia are not yet clear, which complicates their use as biomarkers for assessing tolerance to oxygen deficiency.

The methods for determining the initial tolerance to oxygen deficiency are limited by the need for expensive equipment and highly qualified specialists. In addition, the height of the ascent, the time and methods for achieving it, and the frequency of assessing the severity of AMS vary among hypoxia studies. Duration and severity of hypoxia exposure can be standardized in experiments on laboratory animals. It is necessary to continue research aimed at finding biomarkers of hypoxia resistance, without the direct impact of hypoxia on the organism. In addition, the study of the interconnection between the identified AMS predictors and the molecular response to hypoxia will inform the development of new approaches for personalized prevention, treatment and diagnosis orf inflammatory diseases and malignant tumors and, accordingly, improve the quality of life for these patients.
