# Supplementary material for: Physiological and molecular mechanisms of tolerance to hypoxia and oxygen deficiency resistance markers
Source: Front Mol Biosci. 2025 Nov 26;12:1674608. doi: 10.3389/fmolb.2025.1674608 (PMC12689373; doi:10.3389/fmolb.2025.1674608)
Supplement: Supplementary file 1 [file Table1.pdf]

Supplementary Material

Supplementary Table 1. Physiological markers of hypoxia tolerance

Subjects, (number, sex)	Age (years)	Hypoxic exposure	AMS diagnosis	Method	Tolerance to hypoxia biomarker	AUC	DSe, %	DSp, %	Result of tolerance marker assessment	Reference
106 (106 M)	20.0 ± 3.0	Step-by-step ascent by bus for 7 days (about 40 hours of ascent and about 40 hours of rest and sleep without altitude change) from 400 m to 4100 m	Once after reaching altitude using LLS (33 AMS+, 73 AMS-)	Speckle-tracking echocardiography (analysis of a set of natural acoustic echoes from points with stable visualization during systole and diastole)	MV TMADlateral	n/d	72.7	60.3	Relative weak longitudinal systolic function before ascent is a AMS predictor	(Ke et al., 2021)
39 (18 F, 21 M)	45.5±12.1	Ascent by airplane from 550 m to 1040 m, then on foot for 7 days to 4844 m.	Daily during the course of reaching altitude using LLS (23 AMS+, 16 AMS-)	CPET	VO_{2max}	0.67	46.7	83.3	VO_{2max} below 49.0 ml/min/kg before ascent correlates with AMS+ during ascent without supplemental oxygen use	(Seiler et al., 2023)
46 (27 F, 19 M)	33.3±7.8	Ascent by airplane from 300 m to 3900 m for 3 hours	The day after reaching altitude using LLS (20 AMS+, 26 AMS-)	Smartwatch Huawei Watch GT Runner	VO_{2max}	0.79	65.0	88.5	VO_{2max} before ascent is statistically significantly lower in the AMS+ group	(Ye et al., 2023)
42 (6 F, 36 M)	34.1 ± 13.4	Climb on foot for 4 days from 23.3 m to 4721 m	Daily during the reaching altitude using LLS (18 AMS+, 24 AMS-)	Smartwatch Huawei WATCH GT3	SpO_2	n/d	n/d	n/d	SpO_2 before ascent is statistically significantly lower in the AMS+ group, with a one unit increase reducing AMS risk by 9%	(Zeng et al., 2024)
18 (7 F, 11 M)	36.0 ± 16.0	Ascent by transport for 4 days from 140 m to 2624 m, then 7 days on foot to 4800 m	Daily during morning and evening the reaching altitude using LLS (3 AMS+, 15 AMS-)	Pulse oximetry overnight	SpO_2	0.8	79.0	70.8	Overnight SpO_2 values at 3850 m correlate with AMS at 4800 m	(Joyce et al., 2024)
45 (23 F, 22 M)	28.7 ± 4.4	Ascent by airplane from 43.5 m to 3650 m	During 5 days at altitude using LLS (36 AMS+, 9 AMS-)	Brain MRI	fALFF	0.78	80.5	77.8	fALFF and Degree centrality from resting-state functional MRI, mainly distributed in the somatomotor network before ascension were statistically significantly lower in the AMS+ group than in the AMS- group	(Zhang et al., 2024b)
					Degree centrality from resting-state functional MRI, mainly distributed in the somatomotor network	0.86	77.8	100.0		
48 (25 F, 23 M)	28.8 ± 4.9	Ascent by airplane from 43.5 m to 3650 m	8 h after reaching altitude using LLS (25 AMS+, 23 AMS-)	3D-pCASL	Cortical CBF in the right PCA	0.82	81.8	91.7	Higher Cortical CBF in the right PCA before ascension correlates with AMS+ in M and LI of CBF in the ACA in F	(Zhang et al., 2024a)
					LI of CBF in the ACA	0.75	85.7	63.6		

AUC – Area Under Curve, DSe – Diagnostic Sensitivity, DSp – Diagnostic Specificity, M – Male, F – Female, AMS – Acute Mountain Sickness, LLS – Lake Louise Scale, n/d – no data, MV TMADlateral – lateral Mitral Valve Tissue Motion Annular Displacement, CPET – Cardiopulmonary Exercise Test, VO_{2max} – maximum oxygen consumption, SpO_2 – blood oxygen saturation, MRI – Magnetic Resonance

References

Joyce, K. E., Ashdown, K., Delamere, J. P., Bradley, C., Lewis, C. T., Letchford, A., Lucas, R. A. I., Malein, W., Thomas, O., Bradwell, A. R., et al. (2024). Nocturnal pulse oximetry for the detection and prediction of acute mountain sickness: An observational study. *Exp. Physiol.* 109, 1856–1868. doi:10.1113/EP091691.

Ke, J., Yang, J., Liu, C., Qin, Z., Zhang, J., Jin, J., Yu, S., Tan, H., Yang, Y., Zhang, C., et al. (2021). A novel echocardiographic parameter to identify individuals susceptible to acute mountain sickness. *Travel Med. Infect. Dis.* 44, 102166. doi:10.1016/j.tmaid.2021.102166.

Seiler, T., Nakas, C. T., Brill, A.-K., Hefti, U., Hilty, M. P., Perret-Hoigné, E., Sailer, J., Kabitz, H.-J., Merz, T. M., and Pichler Hefti, J. (2023). Do cardiopulmonary exercise tests predict summit success and acute mountain sickness? A prospective observational field study at extreme altitude. *Br. J. Sports Med*. 57, 906–913. doi:10.1136/bjsports-2022-106211.

Ye, X., Sun, M., Yu, S., Yang, J., Liu, Z., Lv, H., Wu, B., He, J., Wang, X., and Huang, L. (2023). Smartwatch-Based Maximum Oxygen Consumption Measurement for Predicting Acute Mountain Sickness: Diagnostic Accuracy Evaluation Study. *JMIR Mhealth Uhealth* 11, e43340. doi:10.2196/43340.

Zeng, Z., Li, L., Hu, L., Wang, K., and Li, L. (2024). Smartwatch measurement of blood oxygen saturation for predicting acute mountain sickness: Diagnostic accuracy and reliability. *Digit Health* 10, 20552076241284910. doi:10.1177/20552076241284910.

Zhang, H., Feng, J., Zhang, S. Y., Liu, W. J., and Ma, L. (2024a). Predicting Acute Mountain Sickness Using Regional Sea-Level Cerebral Blood Flow. *Biomed. Environ. Sci.* 37, 887–896. doi:10.3967/bes2024.100.

Zhang, W., Feng, J., Liu, W., Zhang, S., Yu, X., Liu, J., Shan, B., and Ma, L. (2024b). Investigating Sea-Level Brain Predictors for Acute Mountain Sickness: A Multimodal MRI Study before and after High-Altitude Exposure. *AJNR Am J Neuroradiol* 45, 809–818. doi:10.3174/ajnr.A8206.
