# Supplementary material for: Physiological and molecular mechanisms of tolerance to hypoxia and oxygen deficiency resistance markers
Source: Front Mol Biosci. 2025 Nov 26;12:1674608. doi: 10.3389/fmolb.2025.1674608 (PMC12689373; doi:10.3389/fmolb.2025.1674608)
Supplement: Supplementary file 2 [file Table2.pdf]

Supplementary Material

Supplementary Table 2. Molecular markers of hypoxia tolerance

Subjects, (number, sex)	Age (years)	Hypoxic exposure	AMS diagnosis	Method	Tolerance to hypoxia biomarker	AUC	DSe, %	DSp, %	Result of tolerance marker assessment	Reference
124 (124 M)	20-23	Ascent in a decompression chamber for 40 min from 200 m to 4500 m, followed by a 12 h stay	After hypoxic exposure using LLS (75 AMS+, 49 AMS-)	qRT-PCR	Combination of expression levels of microRNAs *hsa-miR-134-3p* and *hsa-miR-15b-5p* in saliva supernatant	0.81	n/d	n/d	The expression levels of these microRNAs before ascension are higher in the AMS- group	(Huang et al., 2019)
87 (87 M)	17-35	Ascent by train for 2 h from 200 m to 3648 m	During 5 days after reaching altitude using LLS (41 AMS+, 46 AMS-)	qRT-PCR	Combination of expression levels of circulating microRNAs *hsa-miR-369-3p*, *hsa-miR-449b-3p* and *hsa-miR-136-3p* in peripheral blood	0.99	92.7	93.5	The expression levels of these microRNAs before ascending are higher in the AMS+ group	(Liu et al., 2017)
604 (604 M)	18-45	Ascent by airplane for 2 h from 500 m to 3700 m	18-24 h after reaching altitude using LLS (320 AMS+, 284 AMS-)	MALDI-TOF MS	SNPs rs675666667 in *EPAS1*, rs3025039 in *VEGFA*, rs7292407 in *PPARA* and rs2153364 in *EGLN1*	n/d	n/d	n/d	Each of these SNPs is associated with the occurrence of AMS symptoms of varying severity in different organ systems. SNP rs675666667 (genotype GG) is at higher risk of mild AMS and mild gastrointestinal symptoms, and rs3025039 (genotype CC) is associated with a lower risk of mild AMS and mild headaches	(Zhang et al., 2020)
53 (53 M)	18-20	Ascent by plane for 4 hours from 1200 m to 4300 m	12-24 h after reaching altitude using LLS (30 AMS+, 23 AMS-)	Proximity extension assay technology (based on the interaction of a DNA-labeled antibody with a protein in the test liquid, washing and subsequent amplification of DNA by PCR), Multiple Reaction Monitoring technology (based on mass spectrometry with stepwise peptide selection), machine learning XGBoost	Combination of PHGDH, UBA1, RBKS, GNA13, IGFBP7, FCN2, CA2 and VSIG4 contents in plasma	0.91	n/d	n/d	Plasma PHGDH content before ascension is 4 times more significant in this AMS prediction model	(Yang et al., 2022a)
40 (40 M)	21-27	Ascent by car from 1400 m to 3700 m, adaptation for 7 days without change of altitude, ascent by car for 10 h to 5000 m	36-48 h after reaching 3700 m and 5050 m using LLS, with subjects who developed AMS at 3700 m and 5000 m considered the group with severe AMS, only at 5000 m considered the group with a moderate course (20 AMS+ (8 severe, 12 moderate), 20 AMS-)	LC-MS, ELISA	Combination of SAP, AAT, LT content in plasma	0.97	96.2	88.9	SAP, AAT, LT content in plasma before ascension is higher in the severe AMS+ group compared to the AMS- group	(Guo et al., 2023)
					Combination of SAP and HSP90-α content in plasma	0.87	88.7	72.3	SAP and HSP90-α content in plasma before ascension is higher in the group with moderate course of AMS+ compared to AMS-	
					Combination of SAP and LT content in plasma	0.88	75.5	94.4	SAP and LT content in plasma before ascension is higher in the group with severe course of AMS+ compared to moderate AMS+	
22 (22 M)	20-29	Ascent by train for 36 hours from 200 m to 3648 m	Overnight during 5 days after reaching altitude using LLS (14 AMS+, 8 AMS-)	Spectrophotometry	Uric acid content in serum	0.82	n/d	n/d	The detected markers are lower in the AMS+ group compared to the AMS- group	(Liu et al., 2023)
				Conductometry	PDW	0.84	n/d	n/d		
				Echocardiography	MVE	0.76	n/d	n/d		
					EF	0.78	n/d	n/d		

18 (5 F, 13 M)	23 ± 6	Ascent by car for 2 hours from 50 m to 4300 m	24 h after reaching altitude using a shortened version of the ESQ (5 AMS+, 13 AMS-)	RNA-Seq, linear Support Vector Machine algorithm	Combination of *HLA-DQB1* , *LOC101927999* , *GAS6* and *TNNT1* mRNA expression levels in peripheral blood mononuclear cells	0.92	100.0	83	The mRNA expression levels of *HLA-DQB1* , *LOC101927999* , and *GAS6* in peripheral blood mononuclear cells before ascension are higher in the AMS- group, and *TNNT1* is higher in the AMS+ group	(Yang et al., 2024)
10 (2 F, 8 M)	24 ± 6	Ascent in a decompression chamber for 15 min from 50 m to 4300 m. After an initial 15 min ascent and a rest period, all volunteers underwent a 3-h walk (40 min and 20 min rest) on the treadmill	24 h after reaching altitude using a shortened version of the ESQ (4 AMS+, 5 AMS-)							
83 (83 M)	21-24	Ascent by train for 34.5 h from 500 m to 3650 m	The first night after reaching altitude using the LLS (42 AMS+, 41 AMS-)	SBP, PEF, LC–MS/MS, machine learning model MI-radialSVM-RFE	Combination of SBP, PEF, ACSL4, IGKV1D-16, F13B, PSAP, PVR, MMRN2, 2-Methyl-1,3-cyclohexadiene, calcitriol, 4-Acetamido-2-amino-6-nitrotoluene, 20-Hydroxy-PGE2 in plasma	0.97	94.0	91.0	SBP, PEF, ACSL4 IGKV1D-16, PVR, calcitriol, 2-methyl-1,3-cyclohexadiene, 4-acetamido-2-amino-6-nitrotoluene and MMRN2 in plasma before ascending higher in the AMS- group, and PSAP, 20-hydroxy-prostaglandin E2 and F13B - in the AMS+ group	(Li et al., 2025)

AUC – Area Under Curve, DSe – Diagnostic Sensitivity, DSp – Diagnostic Specificity, M – Male, F – Female, AMS – Acute Mountain Sickness, LLS – Lake Louise Scale, n/d – no data, qRT-PCR - quantitative Real-Time Polymerase Chain Reaction, MALDI-TOF MS - Matrix Assisted Laser Desorption/Ionization Time Of Flight Mass-Spectrometry, SNPs - Single Nucleotide Polymorphisms, EPAS1 - Endothelial PAS domain-containing protein 1, VEGFA - Vascular Endothelial Growth Factor A, PPARA - Peroxisome proliferator-activated receptor alpha, EGLN1 - Egl nine homolog 1, PHGDH - Phosphoglycerate Dehydrogenase, UBA1 - Ubiquitin-like modifier activating enzyme 1, RBKS - Ribokinase, GNA13 - Guanine Nucleotide-binding Protein subunit alpha-13, IGFBP7 - Insulin-like Growth Factor-Binding Protein 7, FCN2 - Ficolin-2, CA2 - Carbonic anhydrase II, VSIG4 - V-set and immunoglobulin domain containing 4, LC-MS - Liquid Chromatography–Mass Spectrometry, ELISA - Enzyme-Linked Immunosorbent Assay, SAP - Serum Amyloid P-component, AAT - Alpha-1-Antitrypsin, LT - Lactotransferrin, HSP90- - Heat Shock Protein 90 alpha, PDW - Platelet Distribution Width, MVE - Mitral peak E Velocity, EF - Ejection Fraction, ESQ - Environmental Symptoms Questionnaire, HLA-DQB1 - Major Histocompatibility Complex, class II, DQ beta 1, GAS6 - Growth Arrest Specific 6, TNNT1 - Troponin T1, SBP - Systolic Blood Pressure, PEF - Peak Expiratory Flow, LC-MS/MS - Liquid Chromatography-tandem Mass Spectrometry ACSL4 - Acyl-CoA Synthetase long-chain family member 4, IGKV1D-16 - Immunoglobulin Kappa Variable 1D-16, F13B - coagulation factor XIII B subunit, PSAP - Prosaposin, PVR - Poliovirus Receptor, MMRN2 - Multimerin-2, PGE2 - Prostaglandin E2

References

Guo, H., Wang, Q., Li, T., Chen, J., Zhang, C., Xu, Y., Chang, Q., Li, H., Sun, W., Han, R., et al. (2023). Potential plasma biomarkers at low altitude for prediction of acute mountain sickness. *Front. Immunol* . 14, 1237465. doi:10.3389/fimmu.2023.1237465.

Huang, H., Dong, H., Zhang, J., Ke, X., Li, P., Zhang, E., Xu, G., Sun, B., and Gao, Y. (2019). The Role of Salivary miR-134-3p and miR-15b-5p as Potential Non-invasive Predictors for Not Developing Acute Mountain Sickness. *Front. Physiol* . 10, 898.

Liu, B., Huang, H., Wu, G., Xu, G., Sun, B.-D., Zhang, E.-L., Chen, J., and Gao, Y.-Q. (2017). A Signature of Circulating microRNAs Predicts the Susceptibility of Acute Mountain Sickness. *Front. Physiol* . 8, 55. doi:10.3389/fphys.2017.00055.

Liu, B., Xu, G., Sun, B., Wu, G., Chen, J., and Gao, Y. (2023). Clinical and biochemical indices of people with high-altitude experience linked to acute mountain sickness. *Travel Med. Infect. Dis.* 51, 102506. doi:10.1016/j.tmaid.2022.102506.

Li, W., Zhang, M., Hu, Y., Shen, P., Bai, Z., Huangfu, C., Ni, Z., Sun, D., Wang, N., Zhang, P., et al. (2025). Acute mountain sickness prediction: a concerto of multidimensional phenotypic data and machine learning strategies in the framework of predictive, preventive, and personalized medicine. *EPMA Journal* . doi:10.1007/s13167-025-00404-9.

Yang, J., Jia, Z., Song, X., Shi, J., Wang, X., Zhao, X., and He, K. (2022a). Proteomic and clinical biomarkers for acute mountain sickness in a longitudinal cohort. *Commun. Biol* . 5, 548. doi:10.1038/s42003-022-03514-6.

Yang, R., Gautam, A., Hammamieh, R., Roach, R. C., and Beidleman, B. A. (2024). Transcriptomic signatures of severe acute mountain sickness during rapid ascent to 4,300 m. *Front. Physiol* . 15, 1477070. doi:10.3389/fphys.2024.1477070.

Zhang, J.-H., Shen, Y., Liu, C., Yang, J., Yang, Y.-Q., Zhang, C., Bian, S.-Z., Yu, J., Gao, X.-B., Zhang, L.-P., et al. (2020). EPAS1 and VEGFA gene variants are related to the symptoms of acute mountain sickness in Chinese Han population: a cross-sectional study. *Mil. Med. Res* . 7, 35. doi:10.1186/s40779-020-00264-6.
